# Rooted in therapeutics: comprehensive analyses of *Cannabis sativa* root extracts reveals potent antioxidant, anti-inflammatory, and bactericidal properties

**DOI:** 10.3389/fphar.2024.1465136

**Published:** 2024-09-16

**Authors:** Valérie Gagné, Natacha Merindol, Raphaël Boucher, Nathalie Boucher, Isabel Desgagné-Penix

**Affiliations:** ^1^ Department of Chemistry, Biochemistry and Physics, University of Quebec at Trois-Rivières, Trois-Rivières, QC, Canada; ^2^ Plant Biology Research Group, Trois-Rivières, QC, Canada

**Keywords:** phytochemical valorization, mitochondrial membrane protection, bactericidal efficacy, anticholinesterase activity, traditional uses, marijuana

## Abstract

Following the legalization of recreational *Cannabis* in Canada in 2018, the associated waste, including *Cannabis* roots, has significantly increased. *Cannabis* roots, comprising 30%–50% of the total plant, are often discarded despite their historical use in Ayurvedic medicine for treating inflammatory and infectious disorders. This study evaluates the phytochemical and therapeutic properties of *Cannabis* root extracts from a high tetrahydrocannabinolic acid, low cannabidiolic acid cultivar (variety Alien Gorilla Glue). We performed ultra high-performance liquid chromatography coupled with mass spectrometry (UPLC-QTOF-MS) to identify the chemical components of the *Cannabis* roots. Extracts using water, ethanol and acid-base solvents were tested for antioxidant activity through free radical scavenging, metal chelation, and lipoperoxidation inhibition assays. Mitochondrial membrane protection was assessed using flow cytometry with the MitoPerOx probe in THP-1 monocytic leukemia cells. Anti-inflammatory potential was evaluated by measuring interleukin-6 levels in lipopolysaccharide-stimulated THP-1 cells. Bactericidal/fungicidal efficacy against *Escherichia coli*, *Staphylococcus aureus*, and *Candida albicans* was determined using the *p*-iodonitrophenyltetrazolium assay. Additionally, we investigated the anticholinesterase activity of *Cannabis* root extracts, given the potential role of plant alkaloids in inhibiting cholinesterase, an enzyme targeted in Alzheimer’s disease treatments. UPLC-QTOF-MS analysis suggested the presence of several phenolic compounds, cannabinoids, terpenoids, amino acids, and nitrogen-containing compounds. Our results indicated significant antioxidant, bactericidal, and anticholinesterase properties of *Cannabis* root extracts from both soil and hydroponic cultivation. Extracts showed strong antioxidant activity across multiple assays, protected mitochondrial membrane in THP-1 cells, and exhibited anti-inflammatory and bactericidal/fungicidal efficacy. Notably, soil-cultivated roots displayed superior anti-inflammatory effects. These findings demonstrate the remarkable antioxidant, anti-inflammatory, and anti-microbial activities of *Cannabis* roots, supporting their traditional uses and challenging their perception as mere waste. This study highlights the therapeutic potential of *Cannabis* roots extracts and suggests avenues for further research and application.

## Graphical Abstract

**Figure:**
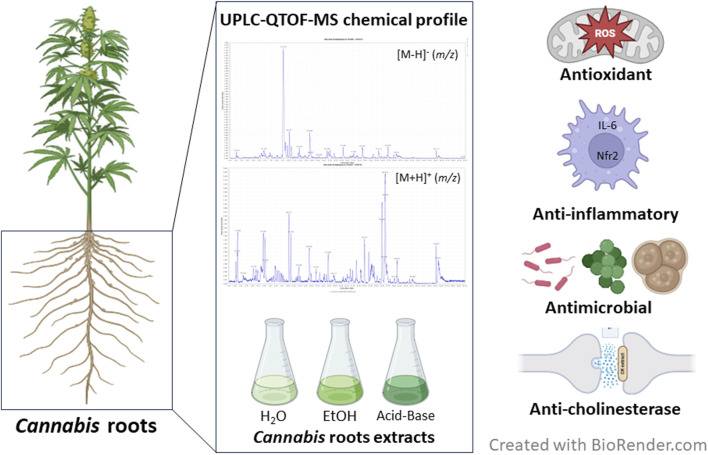

## Highlights

• *Cannabis* roots contain cinnamate, cannabisins, *N*-feruloyltyramine and cannabisativine
• Extracts show potent antioxidant, anti-inflammatory, and antimicrobial activities
• Soil-cultivated roots exhibit superior anti-inflammatory effects
• Extracts inhibit cholinesterases, indicating potential antineurodegenerative effect
• The study confirms traditional uses legitimacy, validating historical therapeutic applications

## 1 Introduction

*Cannabis sativa* is an annual herbaceous plant of the Cannabaceae family (Yang et al., 2013) and its cultivation is believed to have originated from Central Asia (Russo et al., 2008). Besides its fibers, which is thought to be the basis for its domestication, this plant has been used as a source of food and oil, for its medicinal and recreational properties, as well as its role in religious rituals (Piluzza et al., 2013). Many of *Cannabis*’ medicinal properties are attributed to the presence of cannabinoids, a class of terpenophenolic compounds mainly associated to *Cannabis* that has garnered substantial interest in recent years. Two cannabinoids are more abundant and thus more studied, tetrahydrocannabinol (THC) and cannabidiol (CBD), and they exert their effects on humans through the endocannabinoid system (Amin and Ali, 2019). It is in great part due to the growing interest in these compounds that the agricultural landscape surrounding this plant has evolved in recent years.

The traditional uses of *Cannabis* roots for therapeutic properties traces back to ancient times, with its earliest mentions found in the Chinese pharmacopeia attributed to Emperor Shen Nung (Gagné et al., 2024). In it, he advocated for the use of female *Cannabis* plants to address conditions such as malaria, dysentery, constipation, rheumatic pain, attention disorders, and female disorders (Friedman and Sirven, 2017; Rasmusson, 2014). Over time, further applications emerged, encompassing ailments ranging from gout and burns (Russo, 2007) to tumors (Classen, 2001), sore muscles, stiff joints, rheumatism (Rabelais, 1990), menstrual disorders, placental retention, *postpartum* haemorrhage (Bernard, 1938), and skin inflammation (Rasmusson, 2014). These uses were extensively documented in the *Theatrum Botanicum* and The New English Dispensatory. The wealth of historical evidence found in these texts lays the foundation for exploring the therapeutic potential of *Cannabis* roots in contemporary research and medicinal practices.

*Cannabis* cultivation has been on the rise in Canada since its legalization for medical purposes in 2001 (Tattrie, 2019) and for recreational use in October 2018. In 2016, the number of production licenses issued by Agriculture Canada amounted to 55, covering a total cultivation area of 8.7 ha. From this area, 80,535 kg of dried *Cannabis* were produced, generating revenues of $245, 733, 000 (Canada, 2016). An increase is observed from 2016, with a total of 522 authorized *Cannabis* cultivators in Canada, 20, 130, 384 square feet of indoor cultivation and 685 ha of outdoor cultivation in 2021 (Canada, 2021a), and revenues of 14 billion dollars (Canada, 2021b). As a result of the period of illegality from 1923 to 2001 (Tattrie, 2019), very little research was conducted on *Cannabis*. Thus, there is a need for an in-depth examination of this industry, not only regarding the effects of the plant on humans but also regarding optimal cultivation methods and the environmental impacts of *Cannabis* cultivation, as well as its potential in biotechnology.

A critical consideration for all types of cultivation, particularly in the context of climate change, is the environmental footprint, which should aim to be as close to carbon neutral as possible. This involves considering processes both upstream and downstream of the harvest. One approach employed by various industries is waste valorisation, which involves converting cultivation waste into value-added products or reusing residues to establish a circular economy. To reduce the environmental impact of *Cannabis* cultivation, it would be interesting to assess waste materials for their therapeutic potential as to maximize culture utilization, therefore making *Cannabis* a more sustainable crop.

*Cannabis* roots, which represent 30%–50% of the plant biomass, do not contain the psychoactive compound THC in any significant amount, thus they are not regulated under the *Cannabis* legislation in Canada, making them easier to valorise. Historical records suggest that they were primarily used as an anti-inflammatory and antimicrobial agent (Ryz et al., 2017), while other less precise sources have touted them for alleviating various other ailments (Andre et al., 2016). The whole root extract contains several bioactive compounds, such as alkaloids, sterols and cannabinoids, that could act together with synergistic effects (Kornpointner et al., 2021; Slatkin et al., 1971; Gruschow, 2020; Menezes et al., 2021; Slosse et al., 2020). Although studies have explored specific families of molecules, such as terpenoids, within *C. sativa* roots, the properties of the total crude extract remain less understood (Kornpointner et al., 2021; Menezes et al., 2021; Lima et al., 2021).

In this study, we conducted chemical and biochemical tests, including total polyphenol determination, free radicals scavenging, and inhibition of micelle lipoperoxidation to assess the extracts’ antioxidant potential. Subsequently, we carried out tests for mitochondrial membrane integrity and interleukine-6 production in THP-1 cells. In addition, the antibacterial effect of the extracts was assessed and the biological effect of the extracts on cholinesterase activity was evaluated. These tests allowed us to demonstrate that *Cannabis* root extracts grown in soil and in hydroponics have a significant antioxidant effect, as well as potential anti-inflammatory effects in addition to anticholinesterase and fungicidal effects.

## 2 Materials and methods

### 2.1 Plant materials

Seeds from *Cannabis. sativa* L. Alien Gorilla Glue strain of the genetics Alien technology x Gorilla Glue #4 with sample’s reported cannabinoids THCA (24%), CBDA (0.7%), and CBGA (not detected) levels and presence of terpenes (caryophyllene, humulene, limonene, pinene, and terpinolene) were purchased (Crop King Seed, Vancouver, Canada), grown and roots were obtained from two medical *Cannabis* users with cultivation licences (MCR-347668 and MCR-185440). The plant name, first described by Carolus Linnæus in 1753, was checked with World Flora Online, wfo id 0000584,001 (WFO, 2024). The roots were derived from vegetative cuttings from the same mother plant. Three plants were cultivated using a hydroponic method, while three others were grown in soil, all under controlled conditions inside a cultivation chamber. In brief, after the vegetative cuttings were taken, the young plants were placed under conditions conducive to vegetative growth (27°C and 50% humidity), with a 24 h light cycle. Flowering was induced by switching to a 12 h light-dark cycle. When approximately two-thirds of the stigmas had turned brown, the flowers were harvested. The roots were cleaned with distilled water, then dehydrated using a food dehydrator (50°C, overnight) before being ground and reduced to powder. This raw material was then used for the extractions.

### 2.2 Extractions

Three different types of extraction were performed: aqueous, alcohol, and acid-base. The proportion of solvent and roots used for each extract were neighboring 10 mL per gram of dried roots. All filtrations were performed by gravity using Whatman paper #5. To mimic traditional phytotherapy methods, the water extraction was conducted in the form of a decoction: the ground roots were macerated in distilled water for 24 h before being gently heated (75°C, 10 min) and filtered. The alcoholic extraction was carried out using continuous solvent extraction (Soxhlet) with ethanol (95%) (Thermo Fisher Scientific, Ottawa, Canada) for a period of 3 hours and 30 min. The acid-base extraction was performed following the method described by de Andrade et al. (2014), with some modifications. The root material was macerated for 24 h in methanol (Thermo Fisher Scientific, Ottawa, Canada) at room temperature and then filtered. The crude extract was acidified with 2% sulfuric acid (Thermo Fisher Scientific, Ottawa, Canada) to a pH of 2 and extracted with chloroform (Thermo Fisher Scientific, Ottawa, Canada) to remove neutral compounds. The aqueous acid solution was basified with 7 N ammonia (Thermo Fisher Scientific, Ottawa, Canada) to a pH of 10, then extracted with chloroform, with said chloroform subsequently evaporated in speedvac to obtain the acid-base extract. All extractions were reported as dry weight and then diluted in a 70% ethanol solution to a final concentration of 5 mg/mL, or in 20% ethanol, with the same concentration (5 mg/mL), for antibacterial and antifungal tests. Extraction yields are reported in the Supplementary Table A1).

### 2.3 Analysis of root extracts chemical composition

The UPLC‐QTOF‐MS analyses were carried out externally by the Centre de Recherche Industrielle du Québec (CRIQ). Briefly, a UPLC analysis was performed using a Waters Acquity Ultraperformance LC system (Waters), equipped with a binary pump system (Waters). An Acquity Ethylene Bridged Hybrid (BEH) C18 column (100 mm 2 mm id, 1.7 mm particle size) from Waters was used. The molecules of the *Cannabis* root ethanol extract were separated with a mobile phase that consisted of 0.2% acetic acid (eluent A) and acetonitrile (eluent B). The flow rate was 0.2 mL/min and the gradient elution was initial, 2% B; 0–1 min, 2%–100% B; 1–30 min, isocratic 100% B; 30–33 min, 100%–2% B; 33–33.5 min, isocratic 2% B; 33–40 min. The mass spectrometry (MS) analyses were carried out on a QTOF Micro mass spectrometer (Waters) equipped with a Z‐spray electrospray interface. Each analysis was performed in both positive and negative mode, and the data were acquired through a mass scan from 100 to 1,250 m/z. The ionization occurred at 120°C using a cone gas flow rate of 50 L/h, desolvation gas flow rate of 350 L/h, and a desolvation temperature set at 200°C. Nitrogen (99% purity) was used as a nebulizing gas. Data interpretation was carried out with the MassLynx 4.1 software. Mass extraction, deconvolution, and isotope and library search were performed using MZMine 2 (Pluskal et al., 2010).

### 2.4 Total phenolic content (TPC)

The TPC quantification was carried out using the Folin-Ciocalteu colorimetric method (Magalhães et al., 2010) with some modifications. In summary, 500 µL of the extracts were mixed with 250 µL of Folin-Ciocalteu reagent (Sigma-Aldrich, Darmstadt, Germany) and vortexed for 3 min. Subsequently, 500 µL of 8% Na_2_CO_3_ (ACROS organics from Fisher, Geel, Belgium) were added, and the volume was adjusted to 2.5 mL with H_2_O. The mixture was incubated for 1 h in the dark at room temperature. Finally, the samples were centrifugated at 1,000 *g* for 5 min at room temperature, the supernatant was transferred to a microplate and absorbance was read at 765 nm on a spectrophotometer (Synergy H1, BioTek). The total polyphenol content was calculated as gallic acid (Thermo Fisher Scientific, Ottawa, Canada) equivalent (Supplementary Figure A1).

### 2.5 ABTS• radical scavenging activity

The assessment of the extracts’ antioxidant capacity was conducted using the method outlined by Re et al. (1999). In summary, the ABTS• radical (2.2′-Azino-bis (3-ethylbenzothiazoline-6-sulfonic acid) (diammonium salt) (Sigma-Aldrich, Darmstadt, Germany) was generated by oxidation with potassium persulfate (MAT laboratory, Québec, Canada) and can be reduced by a hydrogen-donor antioxidant. The reagent consists of a 7 mM ABTS and 2.45 mM potassium persulfate stock, kept at room temperature for 12 h in the dark. The reaction involved mixing 0.2 mL of sample with 2 mL of the ABTS• solution (diluted in ethanol). A_765_ readings were taken at the beginning of the reaction and after 6 min (Synergy, BioTek). Trolox (6-hydroxy-2.5,7,8-tetramethylchromane-2-carboxylic acid) (Sigma-Aldrich, Darmstadt, Germany) was used to build standard curves at the start and end of the reaction and the results are expressed in Trolox equivalents (µM).

### 2.6 Ferric reducing antioxidant power (FRAP)

The ability of the extracts to reduce Fe^3+^ was demonstrated using the FRAP method (Bolanos de la Torre et al., 2015). In summary, a working solution was freshly prepared by mixing 10 volumes of acetate buffer (300 mM, pH 3.6) (Thermo Fisher Scientific, Ottawa, Canada) with one volume of 2,4,6-tripyridyl-s-triazine (TPTZ) (40 mM dissolved in 40 mM HCl) (Sigma-Aldrich, Darmstadt, Germany) and one volume of iron chloride (20 mM in water) (Thermo Fisher Scientific, Ottawa, Canada), warming to 37°C for 10 min before use. Gallic acid (1,000 µM) was employed as a standard. 20 μL of the extracts and the standard were dispensed into the wells of a 96-well microplate, followed by the addition of 280 µL of working solution. The mixture was agitated and incubated at 37°C in the dark for 30 min. Absorbance was measured at 593 nm (Synergy H1, BioTek).

### 2.7 Lipid peroxidation on linoleic acid micelles

Micelles were prepared as follows: 1 g of linoleic acid (ACROS organics from Fisher, Geel, Belgium) was dissolved in 222.72 mL of borate buffer (0.1 M, pH 9, SDS 0.1 M) (Thermo Fisher Scientific, Ottawa, Canada) purged with nitrogen beforehand. The mixture was incubated at 37°C with agitation for 10 min. To assess the appearance of conjugated dienes, 185 µL of 1X Phosphate-Buffered Saline (PBS) was mixed with 2 µL of micelles and 5 µL of sample. 18 μL of an 8 mM solution of 2.2′-Azobis (2-amidinopronane) dihydrochloride (AAPH) (Thermo Fisher Scientific, Ottawa, Canada) was added to each well just before the start of the absorbance reading on the microplate reader. Readings were taken every 5 min over a 5 h period at 234 nm (Synergy H1, BioTek). Ascorbic acid (Sigma-Aldrich, Darmstadt, Germany) was used as a positive control in the same concentration as the extract (5 mg/mL).

### 2.8 Lipid peroxidation of mitochondrial membranes

To measure the antioxidant capacity of the extract in a cellular context, THP-1 cells (American Type Culture Collection [ATCC], Manassas, VA, United States) were exposed to various extracts (1.25 μg/mL) for 24 h at concentration of 2–5 × 10^5^ Cells/mL in 96-wells plate. Subsequently, they were washed twice with PBS. Next, 100 µL of MitoPerOx at 100 nM (Cayman chemical company, Ann Arbor, United States) was added to the wells, followed by an incubation for 30 min. The cells were once again washed twice with PBS, and 500 µM hydrogen peroxide (H_2_O_2_) (Sigma-Aldrich, Darmstadt, Germany) was applied to the cells. After 1 h, the cells were analyzed on a flow cytometry (Cytoflex S, Beckman Coulter). The data was processed with the Flowjo software (v 10.9, BD Biosciences) and the 520/590 mean fluorescence ratio was calculated. This probe specifically binds to the mitochondrial membrane and emits maximum fluorescence at 590 nm. When the membrane is damaged, the emission maximum is shifted to 520 nm. An increase in the 520/590 nm ratio indicates mitochondrial lipid peroxidation.

### 2.9 Cholinesterase inhibition

To assess cholinesterase inhibition, the extracts and standards were diluted to a concentration of 0.125 μg/mL in phosphate buffer (0.1 M, pH 7.5) (Thermo Fisher Scientific, Ottawa, Canada). The reaction buffer consisted of 5.5′-Dithiobis-2-Nitrobenzoic Acid (DTNB) at 0.01 M and the enzyme at 100 U/mL in phosphate buffer at 0.1 M (75 µL per reaction) (Abcam, Waltham, United States). In each well, 75 µL of reaction buffer and 5 µL of sample were added, followed by a 5-min incubation at room temperature. Subsequently, 20 µL of acetylthiocholine iodide at 0.01 M (Abcam, Waltham, United States) were added, the plate was mixed, and absorbance was measured in a kinetic mode every 2 min at 410 nm. Inhibition was calculated using the following formula:

I=100X1−∆i∆e

where Δi represents the absorbance difference between the two readings in the presence of the extracts and Δe is the corresponding difference for the solvent control.

### 2.10 Antibacterial and antifungal capacity

The extracts were tested in 96-well plates at a concentration of 5 mg/mL in 20% ethanol. 50 μL of a 0.5 McFarland unit suspension of the microorganisms to be tested (*Escherichia coli* ATCC 35218, *Staphylococcus aureus* ATCC 6538 and *Candida albicans*) were added. The yeast strain was supplied by the microbiology laboratory of the Université du Québec in Trois-Rivières (Québec, Canada). The plate was incubated for 3 h for bacteria and 6 h for *C. albicans*. Then, 40 µL of *p*-iodonitrophenyltetrazolium (INT; 2.85 g/L) (Sigma-Aldrich, Darmstadt, Germany) were added to the wells, followed by an incubation of 1 h for bacteria and 16 h for *C. albicans*. The wells displaying a yellow color, indicating growth, were spread on agar plates [potato dextrose broth (PDB), potato dextrose agar (PDA), trypticase soy broth (TSB), and trypticase soy agar (TSA)] respectively; Thermo Fisher Scientific, Ottawa, Canada), and the bactericidal/fungicidal activity was evaluated based on the number of colonies observed.

### 2.11 IL-6 production

THP-1 cells were cultivated in complete Roswell Park Memorial Institute (RPMI) medium (Wisent Inc., St-Bruno, Canada) complemented with 10% fetal bovine serum (Wisent Inc., St-Bruno, Canada) and 100 I.U./ml penicillin/streptomycin (Sigma-Aldrich, Darmstadt, Germany) which was refreshed every 2–3 days. The cells were transferred into plates and differentiated into macrophages with 200 nM phorbol 12-myristate 13-acetate (PMA) (Sigma-Aldrich, Darmstadt, Germany) for 24 h. After differentiation, PMA was removed, and the extracts and control were added for a 24 h period. Positive control used was zingerone, a known anti-inflammatory agent, in concentrations of 50 μM. The differentiated THP-1 were stimulated with *E. coli* 0111: B4 lipopolysaccharide (LPS) (Sigma-Aldrich, Darmstadt, Germany) at a concentration of 500 ng/mL for 24 h. The supernatants were collected for enzyme-linked immunosorbent assay (ELISA) for interleukine-6 (IL-6) in accordance with the manufacturer’s protocol (CAT #555220, BD Bioscience, Heidelberg, Germany).

### 2.12 Quantitative real-time polymerase chain reaction (qRT-PCR)

Total RNA was extracted from THP-1 cells using TRIzol reagent (Invitrogen, Carlsbad, United States) and subjected to reverse transcription with M-MLV reverse transcriptase (NEB, Whitby, Ontario) following the manufacturer’s instructions. The relative mRNA expression levels were quantified using the Luna Universal qPCR kit (NEB, Whitby, Ontario). All samples were analyzed in triplicate and normalized using β-actin as the reference gene. The list of primer sequences is available in Table 1.

**TABLE 1:** Primers used for the qRT-PCR. *HO1*: Heme oxygenase 1, *NQO1*: NAD(P)H quinone dehydrogenase 1, *COX-2:* cyclooxygenase 2, *iNOS*: inducible oxide nitric synthase.

Gene	Primer sequences	
*HO1*	Forward	TCT​TGG​CTG​GCT​TCC​TTA​C
	Reverse	CAT​AGG​CTC​CTT​CCT​CCT​TTC
*NQO1*	Forward	GGG​ATG​AGA​CAC​CAC​TGT​ATT​T
	Reverse	TCT​CCT​CAT​CCT​GTA​CCT​CTT​T
*COX-2*	Forward	TAC​TGG​AAG​CCA​AGC​ACT​TT
	Reverse	GGA​CAG​CCC​TTC​ACG​TTA​TT
*iNOS*	Forward	GTC​AGA​GTC​ACC​ATC​CTC​TTT​G
	Reverse	GCA​GCT​CAG​CCT​GTA​CTT​ATC

### 2.13 Cell viability

To determine the optimal concentration of each extract and of the treatments (PMA, LPS and H_2_O_2_), THP-1 cell viability was assessed using the Cell Proliferation Kit I (MTT) (Roche, Mississauga, Canada). MTT assay readings were performed at 575 nm using the plate reader (Synergy H1, Biotech).

All determinations were performed in triplicate. Statistical comparisons between means were performed with Dunnett test at *p* < 0.05. Correlations between different parameters were considered significant at r > 0.95 (*p* < 0.05) and were performed with the GraphPad software (V 10.0.0, by Dotmatics).

## 3 Results

### 3.1 Chemical composition of root extracts

The chemical complexity of Cannabis arises from its extensive array of constituents and their potential interactions, encompassing diverse classes such as phenolics, terpenes, sugars, amino acids, hydrocarbons, and nitrogen-containing compounds (Andre et al., 2016). In this study, the chemical composition of *Cannabis* roots was analysed using untargeted UPLC-QTOF-MS metabolite profiling (Supplementary Material 1). Both positive (M + H) and negative (M−H) ionizations modes were employed to ensure comprehensive detection and identification of compounds during the analysis. A total of 533 ion masses were detected, with a relatively lower count observed in negative ion mode (256) compared to positive ion mode (277), and putative identities of compounds were ascribed to the detected masses (Supplementary Material 1). The results are summarized in Table 2, which provides an overview of representative identified compounds classified into distinct metabolite classes. Notably, phenolics compounds were seemingly more abundant than any other class of metabolite reported in Table 2. Furthermore, a range of terpenoids, including monoterpenes (e.g., menthone), diterpenoids (e.g., gibberellins), and triterpenoides (e.g., steroids) were identified (Table 2). Cannabinoids (e.g., cannabidiolic acid), amino acids (e.g., valine, histidine), and carbohydrates (e.g., glucose) were detected in relatively smaller numbers. Also, various nitrogen-containing compounds such as alkaloids (e.g., cannabisativine) and lignanamides (e.g., cannabisin D, E, F and G) were detected (Table 2). All in all, the chemical analysis of *Cannabis* roots revealed a diverse array of metabolites, underscoring the intricate and multifaceted chemistry inherent in *Cannabis*.

**TABLE 2:** Chemical composition of ethanol extracts of *Cannabis* roots using UPLC-QTOF-MS analysis in positive (M + H) and negative (M−H) ionization mode.

Putative identity^a^	RT^b^	*m/z* ^c^	*m/z* ^d^	ppm^e^
	(min)	[M + H]	[M-H]	Error
Phenolic compounds				
Aromatic aldehyde	1.42	107,0488		665
Catechol	35.51	111,0348		695
4-Nitrocatechol	0.92	155,9887		777
1-Phenylethylamine	35.15	122,0954		717
Phenylpyruvate	1.42	165,0788		492
3-(3,4-Dihydroxyphenyl)lactate	35.48	199,0341		4,411
Salicylate	35.51	139,0196		736
*trans*-Cinnamate	22.37	149,0283		883
4-Dodecylphenol	25.45	263,2641		516
4-Hexyloxyphenol	17.73		193,1517	612
Phenylacetaldehyde	10.13		119,046	873
D-Prephenyllactate	10.47		297.09	505
*N*-Feruloyltyramine	10.48		312,1701	416
Chalcone	16.18		207,1309	623
Sinapate	8.01		223,0901	537
Frutinone A	9.03		263,049	688
Bruceine D	9.79		409,1988	491
Gossypol	6.84		517,2145	671
Isobutrin	10.05		595,1918	518
Verbascoside	13.58		623,1482	725
Parishin B	17.01		727,1942	558
Cinnamtannin B1	9.08		863,2094	626
Cannabisin D	11.82		623,2086	788
Cannabisin F	13.59		623,2086	788
Cannabisin G	13.60		623,2809	672
Cannabisin E	11.78		641,2403	716
Cannabinoids				
Cannabidiolic acid	5.80	359,1983		839
8-Hydroxycannabinol	35.11		325,1789	679
Cannabielsoin	13.54		329,2262	831
Terpenoids				
(−)-Menthone	2.57	155,1198		839
(+)-Isomenthone	4.17	155,1198		839
(−)-Menthol	6.11	157,1389		770
Gibberellin A1	6.33	349,2003		572
Gibberellin A3	7.67		345,127	790
3-(O-Geranylgeranyl)-sn-glycerol 1-phosphate	5.58		443,213	647
(2E,6E)-Farnesol	16.35		221,1454	1,015
3beta-Hydroxysteroid	21.79	277,2379		945
16alpha-Hydroxysteroid	18.76		291,2136	983
16-Dehydroprogesterone	20.45		311,1633	760
17 alpha-Hydroxyprogesterone	15.15		329,2262	831
14-Demethyllanosterol	9.10		411,3915	749
9-*cis*-10′-Apo-beta-carotenal	5.81	377,2832		839
Terpendole C	7.90	520,3459		680
Amino acids				
L-Valine	14.79	118,0661		710
5-Methylcytosine	6.14	126,0999		239
*N* (pi)-Methyl-L-histidine	7.01	170,1398		236
L-Histidine	4.10		154,0781	466
L-Phenylalanine	2.80	166,1248		392
L-Tyrosine	35.46	182,0415		815
L-Tryptophan	4.81		203,0821	679
5-Hydroxy-L-tryptophan	20.53	221,1305		405
N2,N5-Dibenzoyl-L-ornithine	5.82	341,191		612
Arg-OEt	3.45		201,1309	592
N2-Succinyl-L-arginine	5.70		273,1453	456
Nitrogen-containing compounds				
Benzoylagmatine	20.59		233,1476	653
Guanosine	10.13		282,099	500
Xanthine	35.65	153,0119		641
(−)-Solenopsin A	23.73	254,252		974
Sinapine	5.82	311,1743		596
Aurachin D	7.83	364,3055		534
Aurachin B	4.82	380,3059		510
*p*-Coumaroylagmatine	20.53	277,1895		507
*N*-Caffeoylputrescine	17.79	251,1754		456
Cannabisativine	6.11	382,3088		761
Carbohydrates				
2-Oxoglutaramate	5.84	146,0547		6,510
D-Ribose	1.35		149,0332	649
D-Glucose	8.92		179,0654	528
D-Gluconic acid	0.31	197,0188		716
D-Mannitol 1-phosphate	7.54		261,0463	397
Others				
L-Pipecolate	6.086	130,053		822
Anthranilate	17.73		136,0826	7,050
2-Amino-9,10-epoxy-8-oxodecanoic acid	5.84	216,1228		588
2-Hydroxy-6-oxo-6-phenylhexa-2,4-dienoate	35.56	219,0561		3,923
(6Z,9Z,12Z)-Octadecatrienoic acid	20.95	279,2555		517
(9Z)-Octadecenoic acid	28.57		281,2474	897
(9Z)-(13S)-12,13-Epoxyoctadeca-9,11-dienoic acid	17.74		293,1714	779
Stearolic acid	35.09		279,2249	627
Ursolic acid	25.35		455,3511	766
Linoleate	26.87		279,2249	627
Limonoate	10.44		505,1847	624

^a^ Putative compounds were proposed on the basis of mass spectrometry analyses in comparison with databases using the MZMine, 2.0 analysis software.

^b^ RT, retention time.

^c^ m/z, Mass to charge ratio, represents exact mass from positive ionization mode analysis.

^d^ m/z, Mass to charge ratio, represents exact mass from negative ionization mode analysis.

^e^ ppm error, calculation on the difference between experimental m/z, from theoretical m/z: |(theoretical–experimental)/theoretical|*106.

### 3.2 Total polyphenol content and antioxidant capacity

**FIGURE 1:**
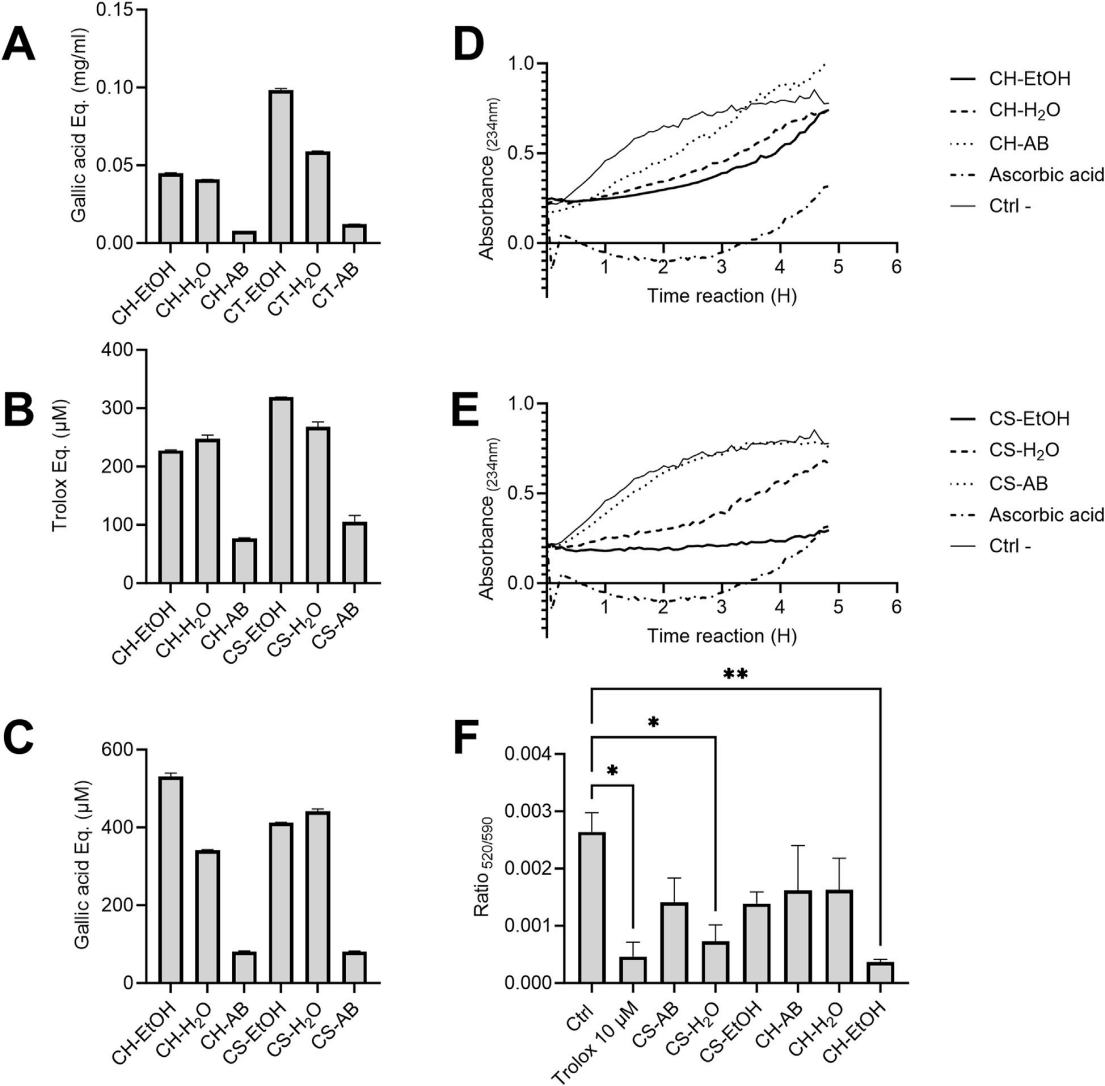
Antioxidant capacity of *Cannabis* root extracts. **(A)** Total phenolic compounds concentration in *Cannabis* root extracts, expressed in milligrams of gallic acid equivalent per millilitre (mg/mL). **(B)** Antioxidant capacity of different *Cannabis* root extracts estimated using ABTS radical neutralization. The *y*-axis represents the difference in Trolox equivalent (µM) between the reading at time 0 and after 6 min of reaction. **(C)** Ferrous ion-reducing capacity of different extracts represented in gallic acid equivalent. In A, B and C, results are presented as the mean ± SD (n = 3). **(D,E)** Monitoring of the formation of conjugated diene systems induced by AAPH in the presence or absence of *Cannabis* root extracts (30 μg/mL). OD_234nm_ measurements were taken every 5 min for a 5 h period. (n = 3). **(F)** Difference in the absorbance ratio (520/590) of the MitoPerOx probe on THP-1 cells subjected to oxidative stress. All extracts were used at a concentration of 1.25 μg/mL, n = 10,000 events, repeated three times. The *p*-value was calculated by one-way ANOVA followed by Dunnett *p < 0.05 **p < 0.01. CH = hydroponic culture, CS = soil culture, EtOH = ethanol extraction, H_2_O = Water extraction, AB = acid-base extraction.

Phenolic compounds such as polyphenols are vital plant specialized metabolites known for their protective role against UV radiation and potential in reducing the risk of age-related diseases. In this study, we analysed extracts derived from the roots of plants cultivated hydroponically (CH) and in soil (CS), all originating from the same mother plant. The extracts were obtained using water (H_2_O), ethanol (EtOH), and an acid-base protocol (AB). The quantity of phenolic compounds present in the six extracts was determined using the Folin-Ciocalteu method (Figure 1A). Notably, ethanol extractions exhibited the highest concentration of polyphenols, followed by water and AB extractions, irrespective of the cultivation method. Furthermore, extracts from soil-cultivated plants generally displayed slightly elevated concentrations of phenolic compounds with increases of 54% for alcohol extraction, 30% for water extraction, and 35% for acid-base extraction compared to hydroponic cultivation (Figure 1A).

To assess the antioxidant capacity of the extracts, the ABTS radical neutralization assay was employed, measured in Trolox equivalent (µM). Results indicated that CS-EtOH and CH-H_2_O extracts exhibited the highest neutralization capacity, while the acid-base extracts demonstrated the lowest efficacy, correlating with their total polyphenol profiles (Figure 1B). No significant neutralization capacity was observed in the solvent-only controls. Additionally, the antioxidant capacity, through the ferrous ion-reducing capacity assay, calculated in gallic acid equivalent (µM), was evaluated (Figure 1C). It is interesting to note that the pattern observed mirrored that of the ABTS test, with ethanolic extracts and aqueous extracts displaying superior antioxidant capacity compared to the acid-base extractions. These findings suggest a pivotal role of phenolic compounds in determining the antioxidant capacity of the extracts.

### 3.3 Lipid peroxidation inhibitory activity

Next, we assessed the membrane protection provided by the various extracts using linoleic acid micelles. When added to these micelles, the oxidant AAPH initiates a chain reaction of lipid peroxidation that can be tracked by the appearance of conjugated diene systems, which have a maximum absorbance at 234 nm. Protection against lipoperoxidation, or the decrease in the formation of conjugated diene systems, is correlated to the antioxidant capacity of an extract. The AB extracts did not prevent lipid peroxidation, while the EtOH extracts were the most inhibitory and the H_2_O extracts showed intermediate activity for both hydroponic and soil cultivation (Figures 1D, E). Most extracts, as well as the ascorbic acid positive control, progressively lost their effectiveness over time. In contrast, the CS-EtOH extract maintained a relatively stable protection after 5 h, with an average absorbance inhibition of 60%. The observed reaction kinetics differences between the extracts and ascorbic acid control suggests that they act through different molecular antioxidant mechanisms. As was observed with the two previous antioxidant assays, the profile of lipid peroxidation inhibition of the extracts seems to follow their polyphenol profiles.

Protection against in-cell oxidative stress was measured using the MitoPerOx probe fluorescence before and after the treatment of THP-1 cells with H_2_O_2_ in the presence or absence of the extracts. As expected, the AB extracts from both hydroponic and soil-cultivated plants had non-significant antioxidant effect, as surprisingly did CH-H_2_O and CS-EtOH, while CH-EtOH and CS-H_2_O extracts significantly reduced the impact of oxidative stress on mitochondrial membranes by 86% and 72%, respectively, relative to the solvent control (Ctrl) (Figure 1F). Interestingly, the CH-EtOH extract offered slightly superior protection when compared to the Trolox control (10 µM).

Overall, significant antioxidant activity was observed in aqueous and ethanolic extracts from both soil and hydroponic cultivated plants, whereas acid-base extracts appeared to have limited to no effect. For the TPC, ABTS, and lipid peroxidation tests, soil extracts proved to be more potent, while for the FRAP test, hydroponic extracts exhibited higher activities. Intriguingly, the most promising extracts in the FRAP test (CH-EtOH and CS-H_2_O) coincided with those providing significant protection to mitochondria, as evidenced by results obtained with the MitoPerOx probe.

### 3.4 Anti-inflammatory effect

**FIGURE 2:**
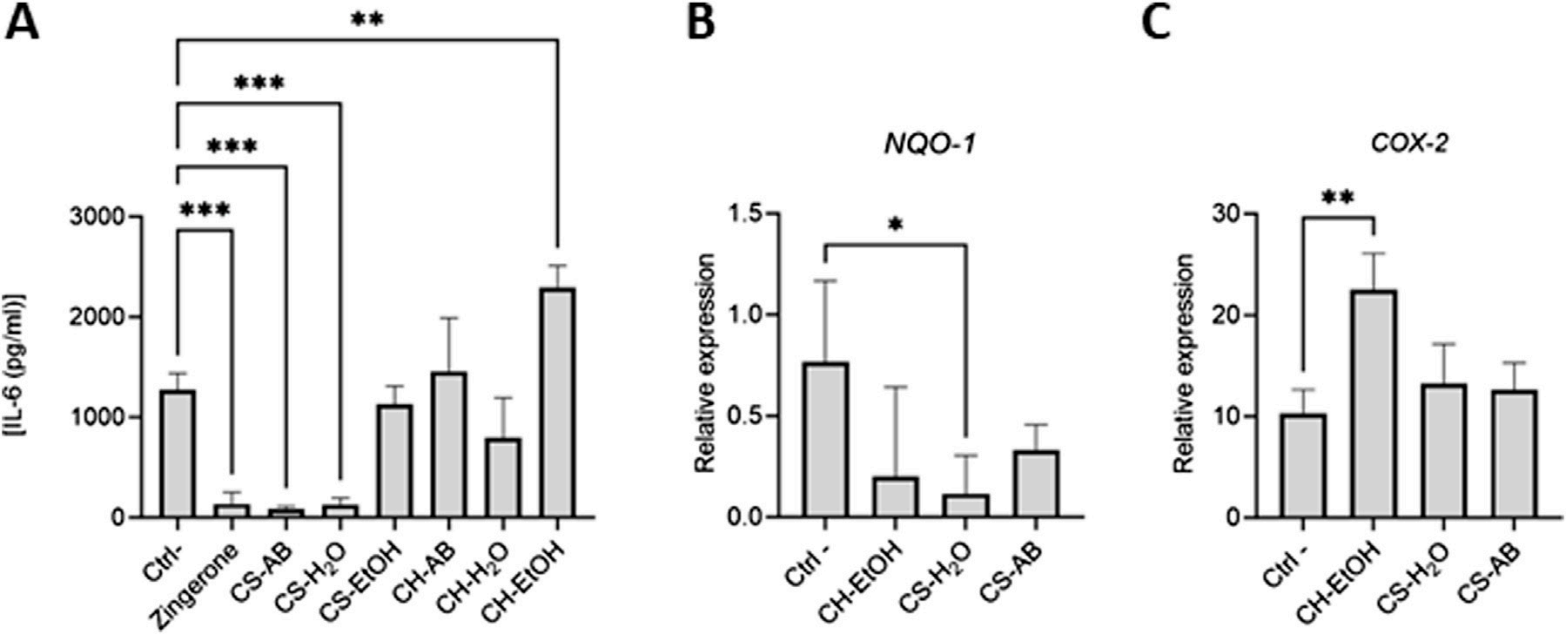
Anti-inflammatory activities of *Cannabis* root extracts. **(A)** IL-6 concentrations in LPS activated differentiated THP-1 supernatants measured by ELISA. Results are presented as mean ± SD (n = 3). **(B,C)** Relative expression of Nrf2-modulated genes in LPS activated differentiated THP-1 cells after exposure to the extracts. Results are presented as mean ± SD (n = 6). Gene expression was normalized using β-actin as reference gene (CH = hydroponic culture, CS = soil culture, EtOH = ethanol extraction, H_2_O = Water extraction, AB = acid-base extraction). The *p*-value was calculated using one-way ANOVA followed by Dunnett with control *p < 0.05, **p < 0.01 and ***p < 0.001.

The anti-inflammatory properties of the extracts were evaluated by measuring IL-6 production after LPS stimulation of differentiated THP-1 cells. Our results revealed a significant decrease in IL-6 concentration in the supernatant of cells treated with CS-AB and CS-H_2_O extracts compared to the control, while there was no significant changed upon treatment with CS-EtOH (Figure 2A). In contrast, extracts from hydroponic culture did not demonstrate any anti-inflammatory effect, with the CH-EtOH extract even exhibiting a significant pro-inflammatory effect under the experimental conditions used. To ensure that the treatments did not compromise the cell metabolic activity, we performed an MTT viability assay (Supplementary Figure A2).

One of the pathways implicated in modulating the inflammatory response of THP-1 cells involves the Nrf2 transcription factor. This pathway, vital for inducing cytoprotective gene expression, serves as the primary defence against oxidative stress (Baird and Yamamoto, 2020). Activation of Nrf2 prompts its translocation into the nucleus, where it regulates the expression of various genes, including NAD(P)H quinone dehydrogenase (*NQO1*), cyclooxygenase 2 (*COX-2*), heme oxygenase (*HO1*), and inducible nitric oxide synthase (*iNOS*) (Saha et al., 2020). Subsequently, RNA extraction followed by qRT-PCR was performed to assess the modulation of Nrf2-dependent gene expression on the samples that had affected IL-6 levels (CS-AB, CS-H_2_O and CH-EtOH) (Figures 2B, C).

It is noteworthy that few significant changes were observed in gene expression levels in response to treatment to *Cannabis* root extracts (Figures 2B, C). Specifically, the CS-H_2_O extract, which reduced IL-6 production, elicited a decrease in *NQ O -1* expression, a gene whose product is involved in the neutralization of reactive oxygen species. Conversely, the CH-EtOH extract, which increased IL-6 production, upregulated *COX-2* expression, suggesting that its pro-inflammatory effect might arise from enhanced production of prostaglandin H2 (PGH2), a COX-2 activity product and precursor to biologically active prostanoids such as various prostaglandins and thromboxane A_2_ (Olszowski et al., 2015). No significant difference in relative expression was detected for *H O -1* and *iNOS* (Supplementary Figure A3).

### 3.5 Cholinesterase inhibitory activity

*Cannabis* roots contain poorly characterized nitrogen-containing compounds including alkaloids, such as cannabisativine (Table 2). Galanthamine, a prominent alkaloid predominately isolated from plants within the Amaryllidaceae family (Harvey, 1995), is renowned for its role in Alzheimer’s disease therapy through the inhibition of cholinesterase enzymes (Sharma, 2019). To expand our understanding of the pharmacological potential of *Cannabis* roots, we investigated this activity across various extracts.

**FIGURE 3:**
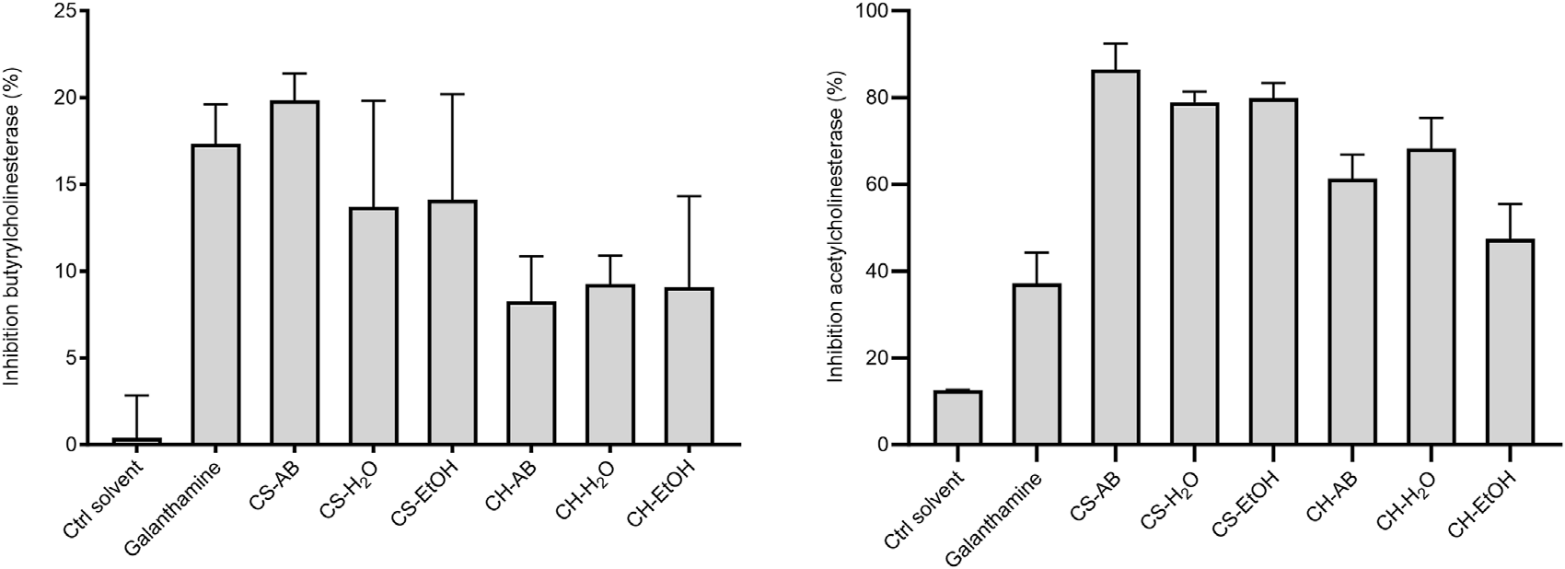
Inhibition of acetylcholinesterase and butyrylcholinesterase by *Cannabis* root extracts and galanthamine as control at a concentration of 0.125 μg/mL Results are presented as the mean ± SD (n = 3). The *p*-value was calculated using one-way ANOVA followed by Dunnett with control (Ctrl solvent) *p < 0.01 and **p < 0.001. (CH, hydroponic culture, CS, soil culture, EtOH, ethanol extraction, H_2_O, Water extraction, AB, acid-base extraction). These findings indicate the presence of molecules with anticholinesterase effects in the extracts, albeit in varying proportions and with different affinities for the two enzymes tested. Although cholinesterase inhibition does not target the underlying cause of Alzheimer’s disease, it remains a recognized therapeutic strategy for alleviating its symptoms (Canada, 2023). Given the escalating burden of Alzheimer’s disease and the observed superior inhibitory activity of *Cannabis* root extracts compared to a known therapeutic agent, it becomes imperative to identify and investigate the candidate molecules responsible for this effect.

The potential cholinesterase inhibition of *Cannabis* root extracts was evaluated using the catalytic reaction of acetylthiocholine iodide by the enzymes acetylcholinesterase and butyrylcholinesterase, with galanthamine serving as a positive control. Regarding the acetylcholinesterase inhibition, five out of the six extracts demonstrated stronger effects (ranging from 45% to 85%) than that of galanthamine (Figure 3). These results suggest that molecules with anticholinesterase effect are present in the extracts, but in varying proportions, and with different affinities for the two enzymes tested. Conversely, for the butyrylcholinesterase, all the extracts displayed a moderate inhibitory activity (ranging from 8% to 20%), akin to that of galanthamine (18%), with extracts from soil cultivation exhibiting greater inhibitory effects compared to those from hydroponic cultivation (Figure 3).

### 3.6 Antimicrobial activity

The antimicrobial activity was assessed using the *p*-iodonitrophenyltetrazolium (INT) colorimetric reduction assay, which measures the metabolic activity of living microorganisms. Briefly, extracts were diluted and incubated with precultured microorganisms. The addition of INT enabled visualization through color changes, with wells exhibiting no color change further examined on agar plates to distinguish between growth inhibition or bactericidal/fungicidal action. Growth results were then correlated with extract concentrations, and inhibitory and bactericidal/fungicidal concentrations were determined accordingly (Table 3).

**TABLE 3:** Results of the antimicrobial activity of *Cannabis* root extracts.

Samples (mg/mL)		*E. coli*		*S. aureus*		*C. albicans*	
		MIC	MBC	MIC	MBC	MIC	MFC
Controls	QAC	0.00351	0.0079	>0.00104	0,00351	>0.00104	0.0016
	EtOH 20%	Eq. > 3	Eq. > 3	Eq. > 3	Eq. > 3	Eq. 1.33	Eq. > 3
Extracts	CH-EtOH	>3	>3	>3	>3	0.89	1.33
	CH-H_2_O	>3	>3	1.33	3	0.89	1.33
	CH-AB	>3	>3	0.89	1.33	0.89	1.33
	CS-EtOH	>3	>3	3	>3	0.26	0.89
	CS-H_2_O	>3	>3	3	3	0.18	3
	CS-AB	>3	>3	1.33	1.33	0.12	3

(CH, hydroponic culture; CS, soil culture; EtOH, ethanol extraction; H_2_O, Water extraction; AB, acid-base extraction, MIC, minimum inhibitory concentration; MBC, minimum bactericidal concentration; MFC, minimum fungicidal concentration; QAC, quaternary ammonium compound. Eq., equivalent, meaning the effect of EtOH; 20% on microorganism is equivalent to the extracts’ effect, in mg/mL).

Our finding indicate that the extracts had no impact on the growth of the Gram-negative bacterium *E. coli*. Conversely, they exhibited inhibitory activity against Gram-positive bacterium *S. aureus*, with inhibitory activity observed at a concentration of 0.89 mg/mL for the AB extraction from hydroponically grown roots (Table 3). Bactericidal activity against *S. aureus* was evident at a concentration of 1.33 mg/mL with both AB extracts.

Remarkably, the extracts displayed substantial inhibitory effects against the fungus *C. albicans*. Indeed, all extracts inhibited its growth at concentrations ranging from 0.12 mg/mL to 0.89 mg/mL, while fungicidal activity occurred at 0.89 mg/mL for CS-EtOH, and at 1.33 or 3 mg/mL for the other extracts (Table 3). These results underscore the antimicrobial effects of *Cannabis* root extracts, albeit with varying effectiveness depending on the microorganism tested and the employed roots’ extraction method.

## 5 Discussion

*Cannabis* roots have a long history of therapeutic use in various cultures around the world, including for their anti-inflammatory, analgesic, and antimicrobial properties (Gagné et al., 2024). Despite this rich traditional use, scientific research on crude extracts properties and safety remains limited. This study aimed to bridge that gap by analyzing the chemical composition of *Cannabis* root extracts and evaluating their biological activities. Given that the relative metabolite profiles can vary significantly between different *Cannabis* strains due to factors such as cultivation conditions, harvest time, and geographical location, we included extracts from both hydroponically and soil-grown samples of the same species.

The UPLC-QTOF-MS analysis revealed a complex array of metabolites. The detection of terpenoids, cannabinoids, amino acids, and nitrogen-containing compounds, including alkaloids like cannabisativine, enriches our understanding of the specialized metabolite profile of *Cannabis* roots. Specifically, phenolic compounds such as salicylate, verbascoside, and frutinone B are known for their antioxidant and anti-inflammatory properties (Amann and Peskar, 2002; Alipieva et al., 2014; Chen et al., 2021). In healthy cells, ROS levels are finely controlled to maintain cellular homeostasis. The main targets of this oxidative stress are lipids, proteins, DNA and RNA, thereby increasing the risks of mutagenesis (Hussain et al., 2016). An imbalance between production and elimination of ROS can lead to chronic inflammation, which is implicated in many chronic diseases. *Cannabis sativa* roots ethanol and water extracts exhibited a strong antioxidant capacity in three different assays (ABTS radical neutralization test, metal chelation test, and protection against lipid peroxidation on linoleic acid micelles). Extracts from soil-grown roots outperformed those from hydroponically grown roots. Soil may represent a more hostile environment, eliciting roots to produce more antioxidants to protect themselves from oxidative stress. The antioxidant capacity aligns with the high polyphenol content and also with the presence of sterols like fucosterol and γ-sitosterol, which are recognized for their ability to neutralize free radicals and protect lipids from peroxidation (Lee et al., 2017; Lee et al., 2003; Javaid et al., 2021; Singh, 2013; Gan et al., 2019; Meinita et al., 2021), and with previous studies (Judžentienė et al., 2023). Phenolic amides, such as cannabisins F, D, and G, could also participate, with recent studies indicating that cannabisin F may exert antioxidant and anti-inflammatory effects via the Nrf2 signaling pathway (Wang et al., 2019).

**FIGURE 4:**
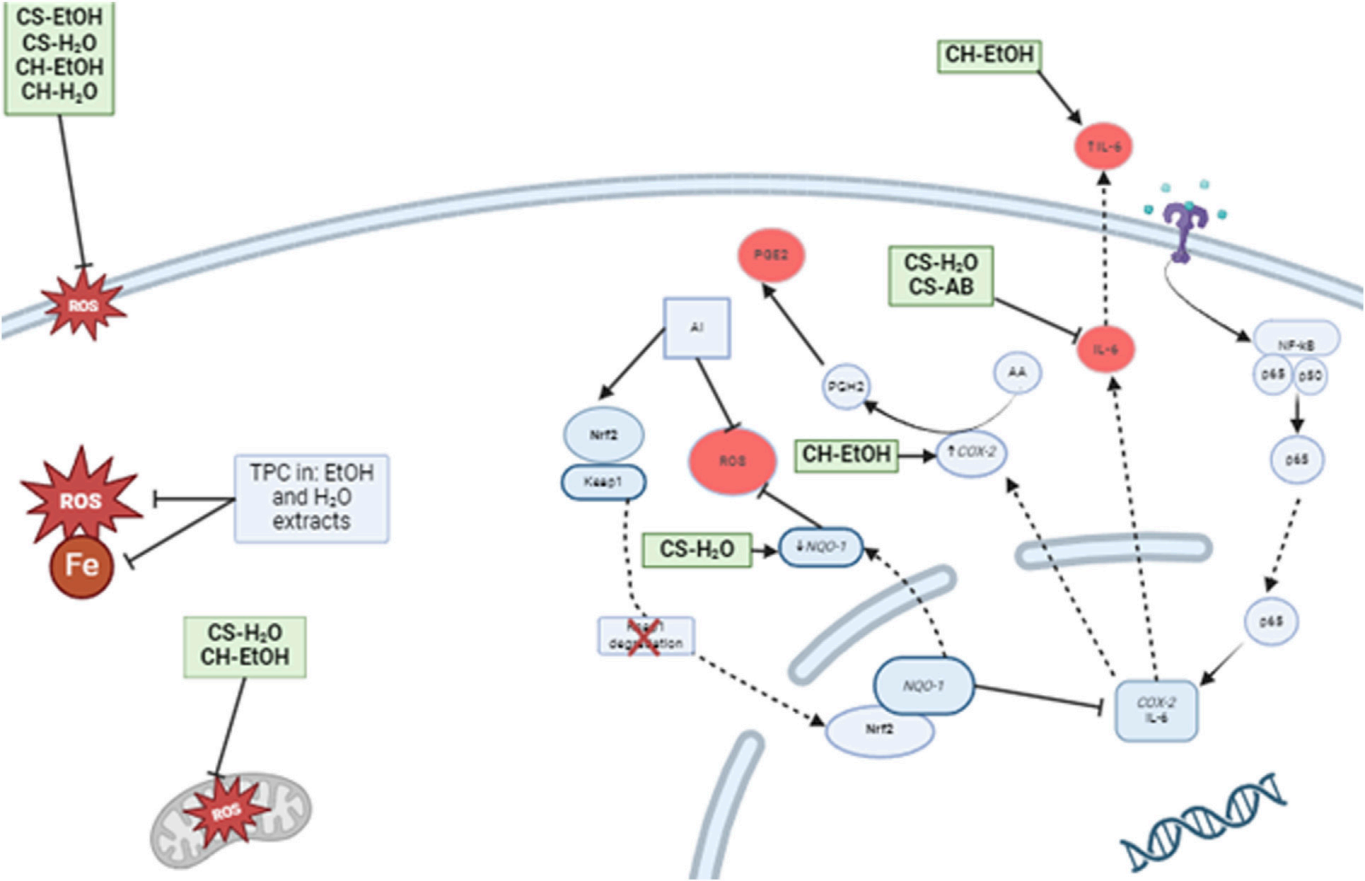
Proposed model of the effects of *Cannabis* root extracts on anti-inflammatory and antioxidant responses of LPS activated immune cells. CS-H_2_O and CS-AB reduced the IL-6 concentration, while CH-EtOH increased it. Additionally, CS-H_2_O was found to decrease the relative expression of *NQ O -1*, while CH-EtOH increased *COX-2* expression. On the cell membrane, the extracts, except AB, inhibited lipid peroxidation, and the total polyphenol content (TPC) of AB extract exhibited radical-scavenging properties and iron chelation. Furthermore, CS-H_2_O and CH-EtOH extracts protected immune cell mitochondria from ROS induced by hydrogen peroxide. (CH, hydroponic culture, CS, soil culture, EtOH, ethanol extraction, H_2_O, Water extraction, AB, acid-base extraction). The figure was created with BioRender.com. In addition to antioxidant activity, we tested the impact of our extracts on inflammation. *In vivo*, an inflammatory state is characterised by the presence of pro-inflammatory cytokines such as TNF-α and interleukin-6 (IL-6). Therefore, it is interesting to observe a reduction in IL-6 production by THP-1 cells in response to two extracts, CS-H_2_O and CS-AB, which do not appear to act through the Nrf2 signalling pathway. Our results are consistent with the study of Lima, (Lima et al., 2021), where an aqueous extract from *Cannabis* roots demonstrated an anti-inflammatory effect in mouse inflammation models, suggesting that the observed effect was related to a reduction in vascular extravasation and inflammatory cells migration. Another study, conducted by (Menezes et al., 2021), demonstrated an anti-inflammatory response in mouse lungs following treatment with an aqueous extract of *Cannabis* roots, with a reduction in the total number of leukocytes in bronchoalveolar fluids. This reduction is also likely related to a decrease in vascular extravasation. Future studies could test this hypothesis by treating endothelial cell cultures with *Cannabis* root extracts, followed by an assessment of the vascular permeability as an indication of extravasation potential. In the realm of anti-inflammatory action, it is interesting to note that the results of our extracts are promising and consistent with literary citations.

Interestingly, while ascorbic acid (the positive antioxidant control) showed diminished lipid peroxidation inhibition over time, the soil EtOH extract maintained its protective effect, suggesting a synergistic action of multiple compounds at different stages of the lipid peroxidation process. This stability indicates that *Cannabis* root extracts might offer a more sustained antioxidant effect, potentially valuable in conditions characterized by chronic oxidative stress, such as neurodegenerative diseases. The MitoPerOx probe was then used to investigate the extracts activity in a biological system. We were able to demonstrate that the CH-EtOH and CS-H_2_O extracts had a protective effect on the mitochondrial membranes of THP-1 cells against oxidative stress *in vivo*, whereas the other extracts were not significatively different from the negative control. Interestingly, the most effective extracts differed between the *in vivo* and *in vitro* oxidation assays, *e.g*., the CH-EtOH and CS-H_2_O only showed intermediate effectiveness on micelles peroxidation prevention while the CS-EtOH extract had the strongest effect. These observations suggest that one or more lipid protective molecules present in the CS-EtOH extract may not have the ability to penetrate inside the cell to directly protect the mitochondria (Figure 4). Alternatively, the isolated cellular model may not fully replicate the complex interactions of a whole organism, potentially affecting the extract’s performance. Issues such as inadequate compound bioavailability, formulation challenges, and differences in cellular metabolism or interactions can also impact effectiveness. These limitations suggest that further research is needed to better understand and address these discrepancies for improved translation of *in vitro* findings to biological systems. While this consideration goes beyond the scope of our study, it would be of significant interest to identify and isolate the metabolites primarily responsible for this effect and to analyse the interactions with various compounds already identified in roots. This could allow the future use of *Cannabis* roots for mitigation of pathologies associated with mitochondrial dysfunction, such as Alzheimer’s disease (Li et al., 2022).

Regarding neuroprotection, the extracts demonstrated significant inhibition of butyrylcholinesterase and acetylcholinesterase, enzymes linked to Alzheimer’s disease. The alkaloids cannabisativine and anhydrocannabisativine, although not fully characterized, may play a role in this inhibitory effect (Bhambhani et al., 2021; Turner et al., 1976). Additionally, the detection of parishin B, traditionally used in Chinese medicine for cognitive enhancement, further underscores the potential health benefits of *Cannabis* root extracts (Li et al., 2016). Given the involvement of oxidative stress in Alzheimer’s disease (Simion and Jurcau, 2019; Teleanu et al., 2019; Jurcau and Ardelean, 2022), the combined antioxidant and cholinesterase inhibitory properties of *Cannabis* root extracts present a compelling case for further investigation into their therapeutic potential for neurodegenerative conditions.

While the extracts did not affect *E. coli* growth in the antimicrobial assays conducted here, they exhibited detectable activity against *S. aureus* and significant effects against *C. albicans*. The presence of β-amyrin and β-sitosterol previously reported (Dan Jin et al., 2020), as well as of gossypol and phenylacetaldhyde detected here, which have documented antimicrobial activities (Keshmiri-Neghab and Goliaei, 2014; Zhu et al., 2011; Kwun et al., 2021; Pierre Luhata and Usuki, 2021), likely contributes to these effects. Moreover, the detection of mollicelin C, a metabolite from plant endophytes with potent antimalarial and antifungal properties (Khumkomkhet et al., 2009; Marcandier, 1758), suggests that the antimicrobial activity of *Cannabis* root extracts might be partly due to interactions between plant metabolites and endophyte-derived compounds. To the best of our knowledge, no previous study had evaluated the antimicrobial action of whole *Cannabis* root extracts.

Finally, the differences in biological activity observed between soil-grown and hydroponically grown roots highlight the influence of cultivation conditions on the metabolite profile and bioactivity of Cannabis roots. Soil-grown roots demonstrated superior anti-microbial, antioxidant and anti-inflammatory activities, while hydroponic cultivation coupled with alcohol extraction appears to have a unique pro-inflammatory effect. This could be due to the presence of symbiotic microbial communities that enhance the plant’s stress responses.

Overall, *Cannabis* roots appear to demonstrate promising antioxidant effects, particularly for water and alcohol extracts. Precisely these extracts are the types of preparations that are described in the ethnopharmalogical reports, adding to the credibility of the ancient medicinal uses of what is, today, considered waste. This research represents the foundation of the exploration of the chemical, biochemical, and biological properties of *Cannabis* roots, a relatively uncharted territory. The results obtained open various potential applications for *Cannabis* root extracts, offering the opportunity to benefit from this, until now, wasted plant material. The observed antioxidant effect could find applications in the cosmetic industry, particularly as an anti-aging agent, or in the food industry to prevent product rancidity. The anticholinesterase effect, with antioxidant activity holds promise in the pharmaceutical field, pending the identification and characterization of suitable candidates. Lastly, the antimicrobial effects warrant exploration in different fields such as in agriculture, where it could be used as an additive to fertilizers for protection against phytopathogens, or in the medical domain for the development of new antibiotics, an ongoing concern in the face of rising multidrug-resistant organisms.

## 6 Conclusion

In conclusion, this study sheds light on the chemical profile and significant therapeutic potential of *Cannabis* root extracts, confirming the validity of their traditional uses and challenging their conventional status as waste products of *Cannabis* cultivation. The results presented in this work add evidence to the broad spectrum of biological systems in which *Cannabis*-sourced derivatives have a potential effect, not only because of cannabinoids, but also because of the possible action of phenolic and nitrogen-containing compounds. Through comprehensive investigation, we have demonstrated their remarkable antioxidant, anticholinesterase, and anti-inflammatory activities, along with their ability to protect mitochondrial membranes. These findings underscore the importance of reevaluating the utilization of *Cannabis* roots in various therapeutic contexts, potentially offering new avenues for drug discovery and development. By recognizing the value of these often-overlooked plant components, we may uncover novel treatments for a range of medical conditions, thereby contributing to the advancement of natural product pharmacology and healthcare innovation. Further research in this area is warranted to elucidate the underlying mechanisms and explore the full therapeutic potential of *Cannabis* root extracts.

## Data Availability

The original contributions presented in the study are included in the article/Supplementary Material, further inquiries can be directed to the corresponding author.

## Supplementary material

Supplementary Data Sheet 1: Supplementary Figures A1, A2, A3 and Table A1.

Supplementary Data Sheet 2: Supplementary Material 1
