# Supplementary material for: Rooted in therapeutics: comprehensive analyses of Cannabis sativa root extracts reveals potent antioxidant, anti-inflammatory, and bactericidal properties
Source: Front Pharmacol. 2024 Sep 16;15:1465136. doi: 10.3389/fphar.2024.1465136 (PMC11440120; doi:10.3389/fphar.2024.1465136)
Supplement: Supplementary file 1 [file DataSheet1.docx]

**Appendix**

**Table A1**. Identification of extracts and yield percentages of different extraction types

| Alien Gorilla Glue strain | Extraction method | Yield % (w/w) |
| --- | --- | --- |
| Hydroponic | Ethanol | 9.2 |
|  | Aqueous | 3.7 |
|  | Acid-Base | 3.2 |
| Soil | Ethanol | 2.1 |
|  | Aqueous | 7.1 |
|  | Acid-Base | 3.3 |

**Figure A1.** Standard curve of the different molecules used to calculate equivalents for antioxidant tests. Each point corresponds to the average of triplicates.

**
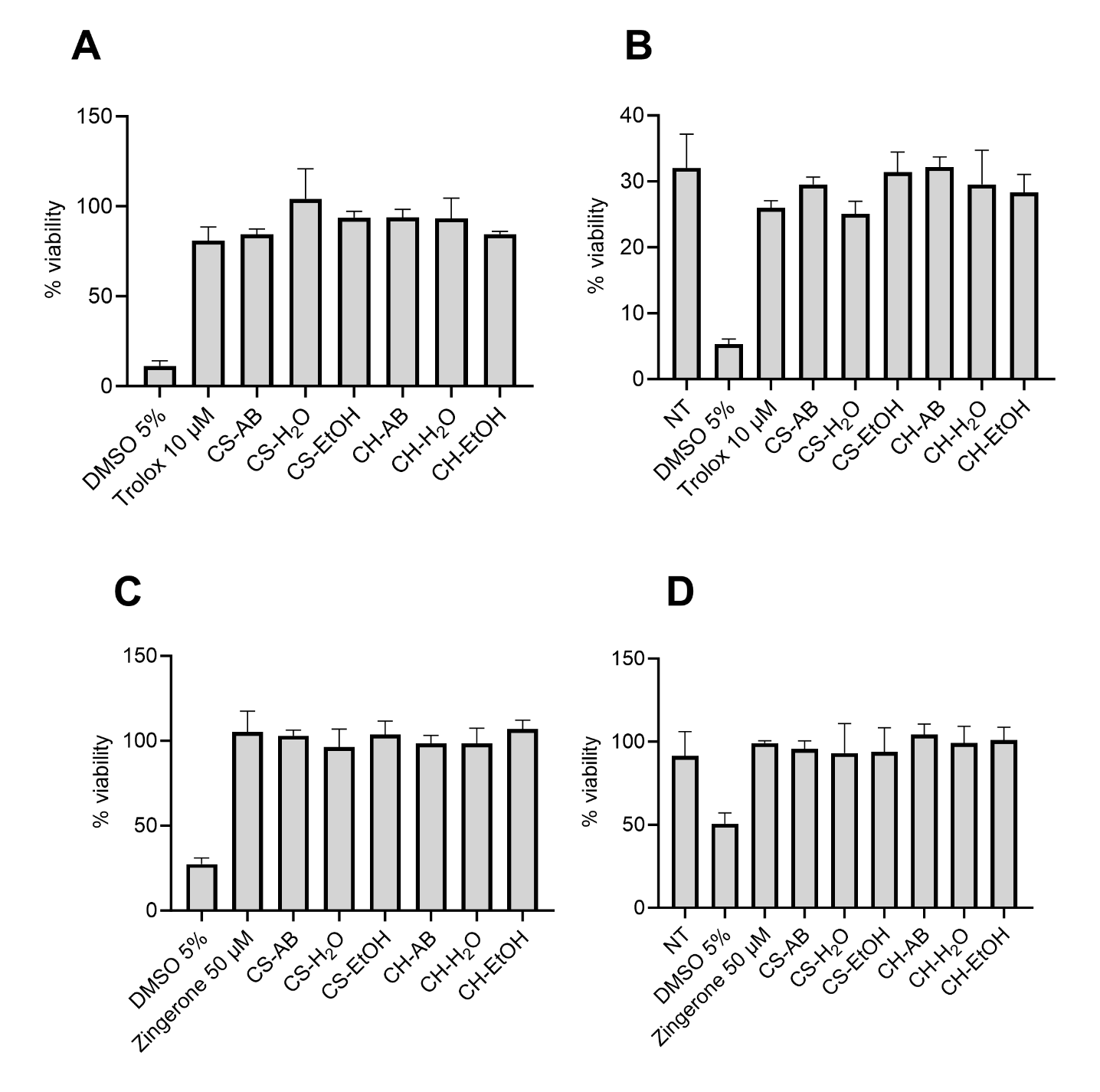
**

**Figure A2.** Viability of THP-1 cells under different experimental conditions assessed using MTT method. A: suspended cells; B: Suspended cells with a 1-hour treatment with 500 µM hydrogen peroxide; C: Cells differentiated with 200 nM PMA; D: Cells differentiated with 200 nM PMA and treated with 500 ng/mL of LPS for 24 hours. Data presented as percentage of control ± SD (n=3). No excess mortality is observed with the extracts or control. Excess mortality is observed with hydrogen peroxide treatment, as expected.


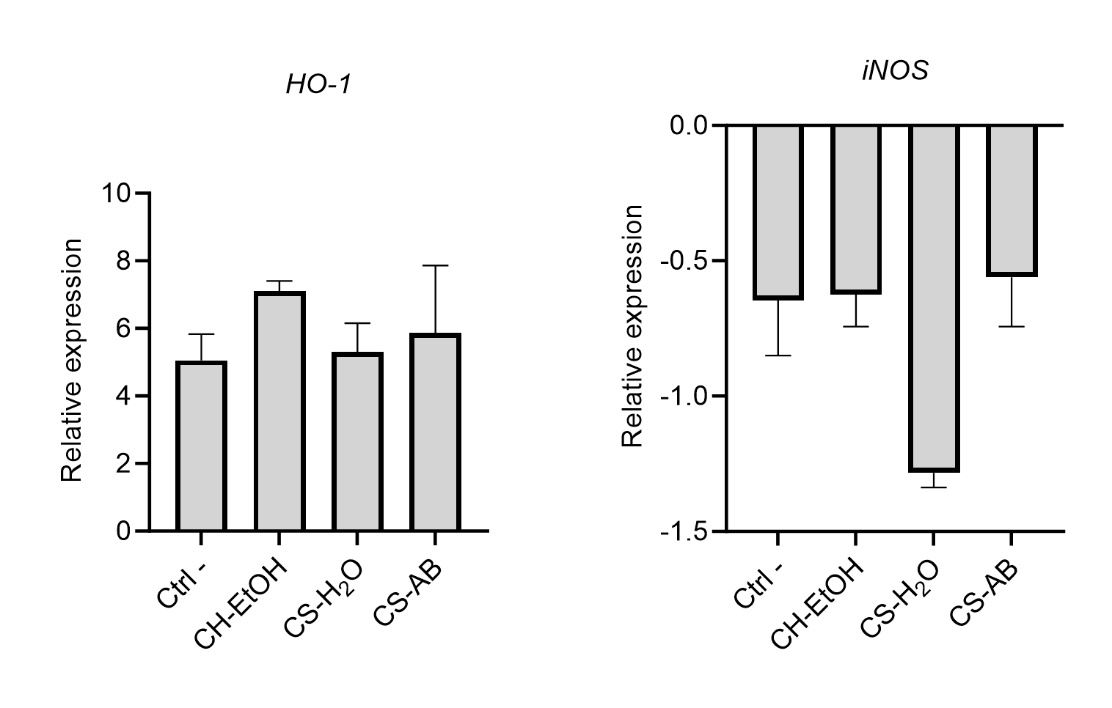


**Figure A3.** Relative expression of Nrf2 target genes in differentiated THP-1 cells after exposure to the extracts and LPS stimulation. Results are presented as mean ± SD (n=6). β-actin was used as the reference gene to calculate relative expression. (CH=hydroponic culture, CS=soil culture, EtOH=Ethanol extraction, H_2_O=Water extraction, AB= acid-base extraction). The P value was calculated by one-way ANOVA with control. No statistical difference was observed.
